# Anticancer Effects and Mechanisms of Berberine from Medicinal Herbs: An Update Review

**DOI:** 10.3390/molecules27144523

**Published:** 2022-07-15

**Authors:** Ruo-Gu Xiong, Si-Yu Huang, Si-Xia Wu, Dan-Dan Zhou, Zhi-Jun Yang, Adila Saimaiti, Cai-Ning Zhao, Ao Shang, Yun-Jian Zhang, Ren-You Gan, Hua-Bin Li

**Affiliations:** 1 Guangdong Provincial Key Laboratory of Food, Nutrition and Health, Department of Nutrition, School of Public Health, Sun Yat-sen University, Guangzhou 510080, China; xiongrg@mail2.sysu.edu.cn (R.-G.X.); huangsy9@mail2.sysu.edu.cn (S.-Y.H.); wusx6@mail2.sysu.edu.cn (S.-X.W.); zhoudd6@mail2.sysu.edu.cn (D.-D.Z.); yangzhj57@mail2.sysu.edu.cn (Z.-J.Y.); saimaiti@mail2.sysu.edu.cn (A.S.); 2 Department of Clinical Oncology, Li Ka Shing Faculty of Medicine, The University of Hong Kong, Hong Kong 999077, China; zhaocn@connect.hku.hk; 3 School of Chinese Medicine, Li Ka Shing Faculty of Medicine, The University of Hong Kong, Hong Kong 999077, China; shangao@connect.hku.hk; 4 Department of Thyroid and Breast Surgery, The First Affiliated Hospital of Sun Yat-sen University, Guangzhou 510080, China; zhyunj2@mail.sysu.edu.cn; 5 Research Center for Plants and Human Health, Institute of Urban Agriculture, Chinese Academy of Agricultural Sciences, National Agricultural Science & Technology Center, Chengdu 610213, China; ganrenyou@caas.cn

**Keywords:** berberine, anticancer, mechanism, bioavailability, safety

## Abstract

Cancer has been a serious public health problem. Berberine is a famous natural compound from medicinal herbs and shows many bioactivities, such as antioxidant, anti-inflammatory, antidiabetic, anti-obesity, and antimicrobial activities. In addition, berberine shows anticancer effects on a variety of cancers, such as breast, lung, gastric, liver, colorectal, ovarian, cervical, and prostate cancers. The underlying mechanisms of action include inhibiting cancer cell proliferation, suppressing metastasis, inducing apoptosis, activating autophagy, regulating gut microbiota, and improving the effects of anticancer drugs. This paper summarizes effectiveness and mechanisms of berberine on different cancers and highlights the mechanisms of action. In addition, the nanotechnologies to improve bioavailability of berberine are included. Moreover, the side effects of berberine are also discussed. This paper is helpful for the prevention and treatment of cancers using berberine.

## 1. Introduction

According to the WHO report in 2020, there were approximately 19.3 million new cancer cases and nearly 10 million deaths, which were mainly attributed to lung, colorectal, liver, stomach, and breast cancers [1]. Under such circumstances, it is urgent to research the prevention and treatment strategies of cancers. The routine treatments for cancers are surgery, radiotherapy, and chemotherapy, but they showed limited actions with some adverse effects. The anticancer effects of natural products have become a research hotspot, due to their low or non-toxicity. Many studies showed that natural compounds from fruits, vegetables, tea, coffee, spices, and medicinal plants could play an important role in the prevention and treatment of cancers with different mechanisms of action [2,3,4,5,6,7,8,9].

**Figure 1:**
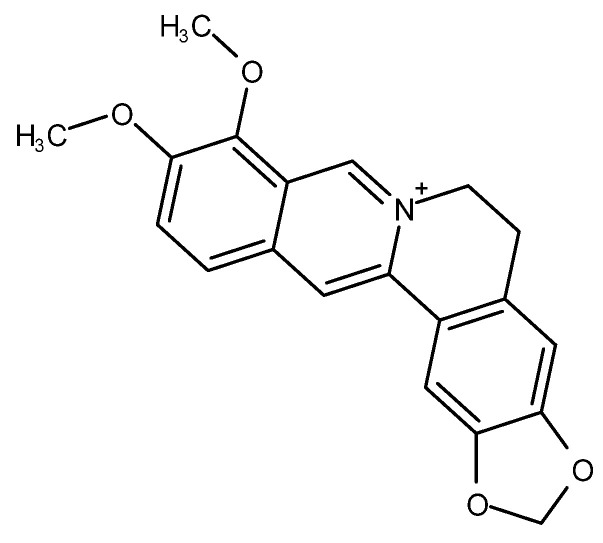
The chemical structure of berberine.

Berberine is a famous natural compound and exists in some medicinal herbs [10,11]. The chemical structure of berberine is shown in Figure 1. Berberine shows many bioactivities, such as antioxidant, anti-inflammatory, cholesterol-lowering, antidiabetic, anti-obesity, and antimicrobial activities [10,12,13,14]. In addition, its anticancer effects and mechanisms have been widely studied, and the results showed that berberine could be a promising agent for the prevention and treatment of various cancers, such as breast, lung, gastric, liver, colorectal, ovarian, cervical, and prostate cancers [15,16,17,18,19]. We collected literature from the PubMed and Web of Science databases since 2016 year. This review summarizes effects and mechanisms of berberine on various cancers, and a special attention is paid to its mechanisms. The bioactivities of berberine, including anticancer activity, have been a research hotspot. Therefore, many review papers on berberine have been published in the literature from different points of view [20,21,22,23]. This paper is an updated review on anticancer effects and the mechanisms of berberine, and it highlights anticancer activity through targeting antibacterial action, including regulating gut microbiota and inhibiting intratumoral microbes. Now, people consider that the compounds from medicinal herbs exhibit anticancer activity by antioxidant and anti-inflammatory mechanisms. We hope that the compounds from medicinal herbs will attract more attention for their anticancer activity by antibacterial mechanism, and this paper could be very helpful.

## 2. Effects of Berberine on Cancers

### 2.1. Breast Cancer

Breast cancer is a threatening malignant tumor worldwide, with the leading morbidity in females [24,25]. Triple negative breast cancer (TNBC) is one of the most aggressive subtypes in breast cancers [26]. Chronic inflammation is closely related to tumor initiation and progression, and both interleukin-6 (IL-6) and tumor necrosis factor (TNF) are the important indicators of tumor-associated inflammation [27]. One study showed that berberine could suppress TNBC by increasing lactate dehydrogenase (LDH) release from TNBC cell lines, and reducing the secretion of proinflammatory cytokines, TNF-α, IL-1α, IL-1β, and IL-6 [26].

The uncontrolled proliferation of cells is one of the main characteristics of cancer. A study showed that berberine inhibited breast cancer cell proliferation and decreased cell viability [28]. Another study showed that high-expressed metadherin was helpful to cancer cell proliferation, while berberine reduced metadherin and inhibited cancer cell proliferation [25]. In addition, berberine induced TNBC cell cycle arrest under low concentration, but did not injure the normal human breast cells [29]. Moreover, berberine inhibited MDA-MB-468 cells proliferation and induced cell cycle arrest at the G_1_/S phase by inhibiting the cell cycle kinase complex cyclin D/cyclin-dependent kinase 4 (CDK4), as well as activating the cell growth inhibitor p38 [30]. Berberine also inhibited MDA-MB-231 cells proliferation and induced cell cycle arrest at the G_2_/M phase by inhibiting the cell cycle kinase complex cyclin A/CDK1 and cell growth-related AKT/ERK pathways [30].

Berberine could suppress breast cancer cell invasion and metastasis through several pathways. For example, berberine could decrease cell migration through downregulating levels of several key chemokine receptors in MCF-7 breast cancer cells, such as chemokine receptor 6 (CCR6) and CCR9 [31]. For MCF-7 and MDA-MB-231 cells, berberine could suppress cell migration by upregulating microRNA (miR)-214-3p and reducing the level of its target secretin (SCT) [32]. Moreover, fibronectin is one of the most abundant extracellular matrix glycoproteins, which is related to cancer cell invasion and metastasis. Berberine could inhibit cell invasion and migration through decreasing fibronectin expression in TNBC cells via inhibiting activator protein 1 (AP-1) activity [33].

Apoptosis could contribute to cancer cell death, which is beneficial to the prevention and treatment of cancers [34]. High-dose berberine (80 µM) directly induced breast cancer cell apoptosis via the AMPK-p53 signaling pathway [35]. Another study showed that berberine inhibited breast cancer cell growth and induced cell death via inducing the nucleolar stress response and upregulating p53 [36]. Moreover, berberine induced breast cancer cell apoptosis and G_2_/M arrest by upregulating miR-214-3p expression [32]. In addition, p21/cip1 and p27/kip1 were important regulators of the cell cycle, while berberine could upregulate levels of p21/cip1 and p27/kip1, as well as increase their nuclear localization and post-translational protein stability via downregulating Akt, and then elevate cell cycle arrest and induce breast cancer cell apoptosis [37].

Berberine could also enhance the anticancer effects of other anticancer drugs. For example, one study revealed that berberine could moderate the X-ray cross complementing group 1 protein (XRCC1)-mediated base excision repair to enhance the sensitivity of cancer cells to chemotherapeutic drugs, such as cisplatin, camptothecin, and methyl methanesulfonate [38]. Another study indicated that the combination of berberine with emodin synergistically inhibited breast cancer cell growth via inhibiting salt-inducible kinases 3 (SIK3) activity and induced G_0_/G_1_ phase cell cycle arrest and apoptosis of breast cancer cells by attenuating Akt signaling [39]. In addition, the combination of berberine with chemotherapeutic drugs (cisplatin and 5-fluorouracil) showed synergistic effects on suppressing breast cancer cell proliferation, thus inducing apoptosis and inhibiting cell migration [40,41]. Anyway, berberine could reverse the multidrug resistance of breast cancer by suppressing the efflux function of ATP-binding cassette transporters [42].

A study comprehensively analyzed the intratumoral microbiome, including breast, lung, ovary, pancreas, melanoma, bone, and brain tumors, and found that each tumor type had a distinct microbiome composition, and breast cancer showed a particularly rich and diverse microbiome [43]. Another study found that tumor-resident microbiota played an important role in promoting metastatic colonization in breast cancer [44]. In addition, *Helicobacter hepaticus* could translocate from the intestine to breast tissues and promote the progression of breast cancer [45]. On the other hand, berberine is widely used as an antimicrobial drug for the treatment of diarrheal infection [46,47] and shows antimicrobial activities against several microorganisms, such as *Helicobacter pylori*, *Candida albicans*, and *Klebsiella pneumoniae* [48,49,50,51]. Therefore, we could speculate that berberine might play the anticancer role via inhibiting intratumoral microbes and regulating gut microbiota.

In brief, the related mechanisms of berberine against breast cancer mainly involve inhibiting inflammation, decreasing cell proliferation, suppressing metastasis, and inducing apoptosis. Moreover, the combination of berberine and chemotherapeutic drugs, such as cisplatin, camptothecin, 5-fluorouracil, and methyl methanesulfonate, has a promoting effect in the prevention and management of breast cancer. In addition, berberine possibly exerts anticancer effect via inhibiting intratumoral microbes and regulating gut microbiota, which should be paid special attention in future studies.

### 2.2. Lung Cancer

Lung cancer is the leading cause of cancer death, and non-small-cell lung cancer (NSCLC) is its most common type [52,53]. Several studies indicated that berberine could inhibit the proliferation of lung cancer cells. For example, berberine suppressed NSCLC cell proliferation and colony formation [54]. Another study showed that berberine inhibited the A549 cells proliferation via matrix metalloproteinase 2 (MMP-2)/Bcl-2/Bcl-2-associated X protein (Bax) and Janus kinase 2 (Jak2)/vascular endothelial growth factor (VEGF)/nuclear factor κB (NF-κB)/AP-1 signaling pathways [55]. Additionally, berberine arrested the cell cycle at G_1_ phase via the Akt/CREB signaling axis and suppressed lung cancer cell proliferation via inhibiting the proliferative kinase signaling [56]. Another study showed that berberine inhibited NSCLC cell growth via suppressing DNA repair and replication both in vitro and in vivo [57]. Anyway, berberine suppressed tumor growth of human NSCLC xenografts in vivo via SWI-independent-3 transcription regulator family member A (Sin3A)/topoisomerase II β (TOP2β) pathway [54]. Moreover, berberine induced NSCLC cell apoptosis by inducing DNA damage via downregulation of TOP2β level [54]. Furthermore, berberine promoted NSCLC cell apoptosis through the miR19a/TF/MAPK signaling pathway [58]. Berberine could also induce NSCLC cell apoptosis via activating the ROS-mediated apoptosis signal-regulating kinase 1/JNK and mitochondrial apoptotic pathways [59].

Berberine could increase the anticancer effect of tyrosine kinase inhibitors against lung cancer. For instance, the combination of berberine with icotinib showed a synergistic inhibiting effect on H460 and H1299 cells by inducing autophagic cell death and suppressing cancer cell migration and invasion [60]. In addition, the combination of berberine and gefitinib synergistically suppressed epithelial-mesenchymal transition (EMT) by regulating the expression of miR-34a-5p and HOX transcript antisense intergenic RNA (HOTAIR) [61]. Berberine could also decrease osimertinib acquired resistance caused by MET gene amplification, therefore enhancing its potency [15].

In short, berberine could prevent and manage lung cancer through several mechanisms, such as inhibiting cell proliferation, inducing apoptosis, and enhancing anticancer activities of tyrosine kinase inhibitors. The related pathways involved MMP-2/Bcl-2/Bax, Jak2/VEGF/NF-κB/AP-1, Akt/CREB, Sin3A/TOP2β, and miR19a/TF/MAPK signaling pathways. More mechanisms of berberine against lung cancer should be explored in the future.

### 2.3. Gastric Cancer

Gastric cancer is one of the most common cancers in the world [62,63]. Several studies showed that berberine could restrain gastric cancer via different pathways. For instance, berberine inhibited SGC-7901 cells proliferation and induced cell cycle arrest at the G_1_ phase [64]. Another study showed that berberine inhibited cancer cell proliferation and reduced IL-8 secretion via deactivating the MAPK signaling pathway in vivo and in vitro [65]. In addition, the epidermal growth factor receptor (EGFR) is overexpressed in gastric cancer and positively related to poor clinical outcomes. Berberine inhibited the activation of signal transducer and activator of transcription 3 (STAT3) by suppressing the phosphorylation of EGFR, subsequently downregulating the expression of apoptosis and cell cycle related proteins, such as Bcl-xL and cyclin D1 [66]. The anticancer potential of berberine could also involve activating autophagy. One study showed that berberine induced BGC-823 cells autophagy via the inhibition of the mTOR, Akt, and MAPK pathways [67]. Anyway, a study revealed that berberine influenced cancer-related pathways via modulating the expression of circular RNA (circRNA) and their corresponding target genes, in such a way to inhibit the gastric cancer progression [68]. Furthermore, berberine could sensitize gastric cancer cells to cisplatin, and the potential mechanism might relate to enhancement of apoptosis, suppression of PI3K/AKT/mTOR signaling, and upregulation of miR-203 expression [69,70]. Berberine also had synergistic effect with erlotinib and cetuximab through enhancing cancer cell apoptosis and cell cycle arrest [66]. Moreover, berberine could increase the anticancer effect of evodiamine (EVO), a natural product, via reducing the upregulation of IL-8 induced by EVO [65].

In general, berberine exhibits good anticancer activity to gastric cancer, and the main mechanisms of action include inhibiting cancer cell proliferation, activating autophagy, and enhancing anticancer activities of other anticancer drugs, such as cisplatin, erlotinib, and EVO. These findings suggest that berberine has potential in the prevention and treatment of gastric cancer.

### 2.4. Liver Cancer

Liver cancer is one of the most common causes of cancer deaths [71,72]. The anticancer effects and mechanisms of berberine on liver cancer have been widely studied. A study showed that berberine suppressed Hep3B and BEL-7404 cell proliferation by inhibiting glutamine uptake via suppressing SLC1A5 (a glutamine transporter) in vitro, and it could inhibit the tumor xenografts growth in vivo and reduce the expression of SLC1A5 [73]. Moreover, berberine in high concentrations could inhibit HepG2 cells proliferation and induce cell cycle arrest at G_1_ phase, and it could arrest HepG2 cell cycle at the S phase in low concentrations [74]. Anyway, berberine upregulated the intracellular ROS level, downregulated mitochondrial membrane potential, and then caused cancer cell apoptosis [74]. Moreover, berberine showed synergistic effect with other antitumor drugs. For example, the combination of berberine and sorafenib synergistically inhibited liver cancer cell proliferation and induced cell apoptosis [75]. Furthermore, berberine could prevent liver cancer by inhibiting the progression of several precursor steps, such as alcoholic fatty liver disease and non-alcoholic steatohepatitis (NASH), through modulating gut microbiota [71,76]. For example, berberine alleviated NASH by increasing the intestinal farnesoid X receptor (FXR) and fibroblast growth factor 15 (FGF15) via modulating gut microbiota, especially increasing the relative abundance of *Clostridiales*, *Lactobacillaceae*, and *Bacteroidale* [77]. Berberine also modulated gut flora, such as increasing the abundance of *Akkermansia muciniphila*, to exert the hepatoprotective effect in alcoholic liver disease [78].

Overall, berberine has a preventive effect on liver cancer by inhibiting the progression of several liver cancer precursor steps via modulating gut flora, and it shows anticancer effects via inhibiting liver cancer cell proliferation, inducing apoptosis, and increasing the anticancer activities of other antitumor drugs.

### 2.5. Colorectal Cancer

Colorectal cancer (CRC) is one of the most common malignant tumors and became the world’s fourth most lethal cancer [79,80]. One study showed that berberine inhibited colon cancer HCT116 and HT29 cells proliferation via decreasing cyclin D1 and increasing p27 and p21, thereby inducing cell cycle arrest at G_1_/G_0_ phase [81]. Another study indicated that berberine inhibited the colon cancer cell proliferation via regulating β-catenin in a dose- and time-dependent manner [82]. Berberine also suppressed β-catenin function by binding a unique region in nuclear receptor retinoid X receptor α (RXRα), and then inhibited colon cancer cell proliferation [83]. Anyway, β-catenin transcriptionally inhibited fat mass and obesity-associated protein (FTO) via binding to its promoter region, and berberine could downregulate m^6^A methylation via decreasing β-catenin and increasing FTO [81]. Moreover, berberine inhibited colon cancer cell proliferation via modulating mitochondrial translation and ribosome biogenesis, as well as promoting calcium mobilization and fat-soluble vitamins metabolism [84]. In addition, CRC proliferation was related to elevating telomerase level and activity. Berberine could downregulate the activity of telomerase in HCT 116 cells and inhibit the CRC proliferation eventually [85]. Berberine could also modulate lipogenesis by targeting the SREBP cleavage-activating protein/sterol regulatory element-binding protein-1 (SCAP/SREBP-1) pathway, and then inhibiting colon cancer cell proliferation [86,87]. Additionally, berberine inhibited colon cancer cell metastasis by suppressing lipogenesis via promoting promyelocytic leukaemia zinc finger (PLZF)-mediated SCAP ubiquitination [88].

A total of 40 μM of berberine induced colon cancer HCT116 and HT29 cells apoptosis [81]. Berberine also suppressed the SW480 cells migration and apoptosis by inhibiting glucose-regulated protein 78 (GRP78) expression and upregulating the cytokeratin expression [89]. For HCT116 cell line, berberine suppressed CRC viability, induced cell apoptosis, activated caspase-3 activity, and downregulated miR-21 expression, as well as promoting the expression of integrinβ4 (ITGβ4) and programmed cell death 4 (PDCD4) protein [90]. Anyway, berberine induced cell apoptosis via modulation of long non-coding RNA (lncRNA)/cancer susceptibility candidate 2 (CASC2)/AU-biding factor 2 (AUF1)/B-cell lymphoma 2 (Bcl-2) axis [91].

Bile acids (BAs) upregulation is a risk factor for colorectal cancer, and berberine downregulated BAs via modulating intestine bacteria [92,93]. Moreover, berberine could exert an anticancer effect by increasing the ratio of *Firmicutes*/*Bacteroidetes*, thus decreasing the abundance of cancer-related bacteria and improving intestinal barrier function [94]. Furthermore, berberine could inhibit the development of azoxymethane/dextran sodium sulfate-induced precancerous lesions, such as crypt destruction, inflammatory cell infiltration, and tumor formation, which indicated that berberine could prevent the occurrence of colorectal cancer [94]. Furthermore, berberine in combination with some other natural products might synergistically promote the anti-tumorigenic properties in CRC. For instance, the combination of berberine and oligomeric proanthocyanidins synergistically induced CRC cell apoptosis via the downregulation of the expression of MYB in the PI3K-Akt signaling pathway [95].

In a word, berberine has inhibitory effects on colorectal cancer, and the main mechanisms involved inhibiting cancer cell proliferation, inducing apoptosis, and modulating intestinal bacteria. Furthermore, most current studies focus on the role of berberine in inhibiting colorectal cancer cell proliferation and inducing apoptosis, while its roles in other anticancer mechanisms, such as suppressing migration, anti-inflammation, and inducing autophagy, need to be further explored in the future.

### 2.6. Ovarian Cancer

Ovarian cancer is a common cancer in women, and a global health problem [96]. The Warburg effect (aerobic glycolysis) is catalyzed by rate limiting enzymes, and provides energy and nutrition to cancer cell proliferation, which helps cancer cells escape from the immune system. Berberine suppressed the Warburg effect by increasing ten-eleven translocation (TET3)-related demethylation and upregulating miR-145, which could inhibit tumor cell proliferation and invasion [97]. In addition, one study showed that transcriptional factor GLI1 aggravated cancer cell migration and cancer stem cell (CSC)-like characteristics, while berberine could downregulate CSC-like characteristics and reverse EMT by inhibiting chemotherapy-activated GLI1/BMI1 signaling pathway [98]. Moreover, the EGFR or ERBB2 overexpression could cause resistance to ovarian cancer cell death and increase tumor-initiating capacity [99]. The evidence suggested that berberine consumed EGFR and ERBB2 in ovarian cancer cells and inhibited the activation of EGFR and ERBB2 downstream targets cyclin D1, MMPs, and VEGF via the EGFR-ERBB2/PI3K/Akt signaling pathway [100].

Berberine could enhance the anticancer effects of other anticancer drugs against ovarian cancer. For example, the combination of berberine and cisplatin synergistically induced ovarian cancer cell death by inducing cancer cell apoptosis via the caspase-dependent and RIPK3-MLKL pathways [101]. Another study showed that berberine could reverse the chemotherapeutic drug VP16-induced repopulation of ovarian cancer cells via blocking the independent phospholipase A_2_ (iPLA_2_)-arachidonic acid (AA)-cyclooxygenase-2 (COX-2)-prostaglandin E_2_ (PGE_2_) pathway and reversing the increased phosphorylation of focal adhesion kinase (FAK) [102]. Berberine could also sensitize ovarian cancer cells to niraparib by inducing oxidative DNA damage and inhibiting homologous recombination repair [103].

In summary, berberine could prevent and treat ovarian cancer by inhibiting cancer cell proliferation, suppressing migration, inducing cancer cell death, and enhancing the anticancer effects of other drugs against ovarian cancer. More anticancer mechanisms of berberine on ovarian cancer should receive attention in the future, such as modulating gut microbiota.

### 2.7. Cervical Cancer

Cervical cancer is the fourth leading female malignancies all over the world [104]. Berberine could inhibit cancer cell viability and suppress tumor growth in cervical cancer. For example, berberine decreased cervical cancer cell viability, inhibited cell migration and invasion, suppressed EMT, and induced cell apoptosis via suppression of keratin (KRT) 17 expression [105]. Moreover, berberine could reduce SiHa cells invasion and migration by reducing transcriptional activities of MMP-2 and urokinase-type plasminogen activator (u-PA), reversing EMT, upregulating E-cadherin, and inhibiting several mesenchymal markers, such as N-cadherin and snail-1 [106]. Additionally, berberine increased GADD153 expression by inducing ROS production, and subsequently led to mitochondria dysfunction followed by activating caspase-3 and cytochrome C release, which then resulted in cervical cancer cell apoptosis [106]. Berberine could also reduce tumor-induced angiogenesis through downregulating VEGF [106]. Furthermore, berberine was a potential adjuvant for other anticancer therapy of cervical cancer. For example, the combination of berberine and matrine synergistically inhibited cervical cancer cell proliferation, triggered cell apoptosis, and induced cell cycle arrest at G1 phase [107]. Berberine could also overcome the radio-resistance caused by low-glucose and hypoxia in the radiotherapy of cervical cancer via regulating the glucose metabolism through PI3K/HIF-1 pathway [108].

Overall, berberine has significant anticancer effect on cervical cancer. The mechanisms of action include decreasing cervical cancer cell viability, inhibiting cell migration and invasion, and inducing cell apoptosis. In addition, berberine could also overcome the radio-resistance to enhance the anticancer effect of radiotherapy.

### 2.8. Prostate Cancer

Prostate cancer is the fifth leading cause of cancer mortality in men [4,109]. A study showed that berberine inhibited cell proliferation and triggered cell apoptosis of 22RV1 prostate cancer cells, and downregulated the expression of androgen receptor (AR), prostate-specific antigen (PSA), COX-2 and Bcl-2, and subsequently inhibited xenograft tumor growth in vivo [110]. Anyway, berberine induced cell apoptosis and inhibited cell proliferation in prostate cancer cell lines via suppressing androgen receptor signaling pathway [111]. Moreover, berberine suppressed the intracellular androgen synthesis via inhibiting the aldo-keto reductase family 1 member C3 (AKR1C3) enzyme activity, and then inhibited prostate cancer cell growth [112]. Berberine also arrested cell cycle at G1 phase and inhibited cell growth in a dose-dependent manner via inhibiting the activation of EGFR [113]. In addition, berberine inhibited prostate cancer cell invasion and migration by downregulating several EMT-related genes, such as platelet-derived growth factor receptor-beta (PDGFRB), collagen, type I, alpha 2 (COL1A2), and bone morphogenetic protein 7 (BMP7) [114]. Furthermore, hypoxia inducible factor-1α (HIF-1α) and its downstream target genes, such as VEGF, could regulate the cell response to hypoxia, and might confer resistance to radiotherapy of prostate cancer. Berberine could increase radiosensitivity of prostate cancer cells through inhibiting the expression of HIF-1α and VEGF, which indicated that it could be an adjuvant in radiotherapy of prostate cancer [115].

Generally, berberine exhibits anticancer activity on prostate cancer both in vitro and in vivo. The mechanisms include inhibiting cancer cell proliferation, suppressing migration, arresting cell cycle, and inducing cell apoptosis. Additionally, berberine could be an adjuvant to enhance the sensitivity of radiotherapy.

### 2.9. Other Cancers

Except for cancers mentioned above, berberine also shows anticancer effects on other cancers, such as pancreatic, bladder, endometrial, esophageal, osteosarcoma, neuroblastoma, and hematopoietic cancers (Table 1). For example, STAT3 was a treatment target for bladder cancer therapy, and the anti-bladder cancer effects of berberine might involve disturbing the AK2-STAT3 signaling pathway via upregulation of miR-17-5p [19]. Anyway, berberine had synergistic effects with several chemotherapeutic drugs of bladder cancer, such as epirubicin and gemcitabine [116,117]. Moreover, an both in vitro and in vivo study showed that berberine inhibited endometrial cancer cell migration via miR-101/COX-2/PGE_2_ signaling pathway [118]. In addition, berberine decreased pancreatic cancer cell viability and inhibited cell migration by regulating citrate metabolism and transportation in cell mitochondria [119]. Berberine could also stimulate hematopoietic cancer cell apoptosis to exert its anticancer effect [120]. Additionally, berberine inhibited esophageal cancer cell growth by arresting cell cycle at G_2_ phase and inducing cell apoptosis, as well as the related mechanisms involved Akt, mTOR/p70S6K and AMPK signaling pathways [121]. Furthermore, the combination of berberine with galangin synergistically inhibited cell growth, arrested cell cycle at G_2_/M phase, and induced cell apoptosis in esophageal cancer cells [122]. Berberine also upregulated p38-MAPK via downregulation of PI3/Akt and Ras-Raf-ERK signaling, and subsequently reversed the EMT in neuroblastoma cells [123]. Another study showed that the combination of berberine and cisplatin synergistically induced cell cycle arrest at G_0_/G_1_ phase and cell apoptosis in osteosarcoma cells via the MAPK pathway [124].

Collectively, berberine showed anticancer effects on different cancers, such as breast, lung, liver, gastric, colorectal, cervical, ovarian, prostate, pancreatic, bladder, endometrial, and esophageal cancers.

## 3. Mechanisms of Action

**Figure 2:**
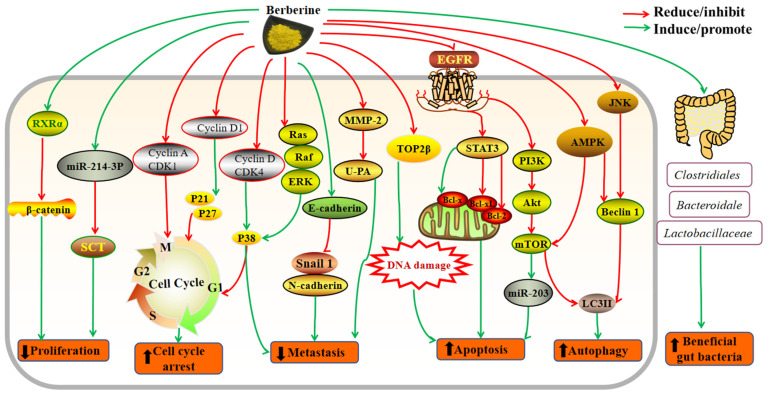
The main effects and mechanisms of berberine on cancers. Berberine could reduce cancer cell proliferation by binding RXRα and subsequently suppressing β-catenin function, upregulating miR-214-3p and reducing SCT; berberine could attenuate cell cycle by inhibiting the levels of CDK1, CDK4, cyclin A, and cyclin D1, and increasing the levels of p21, p27, p38; berberine could inhibit the Ras/Raf/ERK pathway to cause cell cycle arrest and inhibit metastasis; berberine could reduce transcriptional activities of MMP-2 and u-PA to inhibit metastasis; berberine could enhance the levels of E-cadherin, subsequently decrease the level of N-cadherin and snail-1, and ultimately inhibit metastasis; berberine could promote DNA damage through downregulating the level of TOP2β and then induce apoptosis; berberine could promote apoptosis through activation of STAT3 via inhibiting the phosphorylation of EGFR, increasing the expression of Bcl-x, and downregulating the expression of Bcl-xL and Bcl-2; berberine could induce apoptosis by suppressing the PI3K/AKT/mTOR signaling pathway and upregulating the expression of miR-203; berberine could inhibit the PI3K/Akt/mTOR pathway, and enhance the expressions of LC3B-II to promote autophagy; berberine could induce autophagy via JNK/Beclin1 pathway; berberine could regulate gut microbiota by increasing beneficial gut bacteria *Clostridiales*, *Bacteroidale*, and *Lactobacillaceae*. Abbreviations: Akt, protein kinase B; Bcl-2, B-cell lymphoma 2; CDK, cyclin-dependent kinase; EGFR, epidermal growth factor receptor; JNK, c-Jun N-terminal kinase; LC3, microtubule-associated protein light chain 3; mTOR, mammalian target of rapamycin; MMP-2, matrix metalloproteinase 2; PI3K, phosphatidylinositol-3-kinase; RXRα, retinoid X receptor α; SCT, secretin; STAT3, transcription 3; TOP2β, Topoisomerase II β; u-PA, urokinase-type plasminogen activator.

Berberine showed anticancer activity on various cancers, and the mechanisms of action would be summarized below. (1) Berberine could inhibit cancer cell proliferation by upregulating miR-214-3p, reducing the protein levels of its target SCT, regulating β-catenin, downregulating the activity of telomerase, and deactivating the MAPK signaling pathways [32,83,85]. (2) Berberine could arrest cancer cell cycle by increasing the levels of p21, p27, and p38, as well as decreasing the levels of CDK1, CDK4, cyclin A, and cyclin D1 [30,37,81]. (3) Berberine could suppress cancer cell metastasis through reducing transcriptional activities of MMP-2 and u-PA, upregulating E-cadherin, and downregulating several EMT-related genes, such as PDGFRB, COL1A2, and BMP7 [33,106,114]. (4) Berberine could induce cancer cell apoptosis via AMPK-p53, PI3K/AKT/mTOR, miR19a/TF/MAPK signaling pathways, and modulating CASC2/ AUF1/B-cell/Bcl-2 axis [35,58,69,70,91]. (5) Berberine could activate cancer cell autophagy via modulating the MAPK signaling pathway and inhibiting the accumulation of microtubule-associated protein light chain 3II (LC3II) [67]. (6) Berberine could also exert anticancer effects by regulating gut microbiota, such as increasing the ratio of *Firmicutes*/*Bacteroidetes*, increasing the relative abundance of *Clostridiales*, *Lactobacillaceae*, *Bacteroidale*, and *Akkermansia muciniphila* [77,78]. (7) Berberine improved effects of antitumor drugs, such as cisplatin, 5-fluorouracil, doxorubicin, niraparib, icotinib and osimertinib, as well as increased sensitivity of radiotherapy [40,41,60,70]. In brief, the anticancer mechanisms of berberine mainly include inhibiting cancer cell proliferation, suppressing metastasis, inducing apoptosis, activating autophagy, regulating gut microbiota as well as improving the effects of other cancer therapies (Table 1, Figure 2).

## 4. Bioavailability of Berberine

Berberine could inhibit the initiation and progression of many cancers. However, its poor oral bioavailability and low water solubility could reduce its anticancer activities. Moreover, the administration of berberine by intra-muscular and intra-venous could induce anaphylactic reaction [128,129]. Therefore, various strategies have been developed to improve berberine bioavailability and enhance its anticancer activities. These techniques mainly include diverse novel drug delivery systems. For example, a study showed that berberine-loaded liquid crystalline nanoparticles (LCNs) could overcome the poor water solubility and improve the bioavailability of berberine [130]. Another study showed that berberine-loaded bioformulation of silver nanoparticles (AgNPs) showed enhancing cytotoxicity against breast cancer cells and decreasing drug loss during their circulation in the blood [128]. Moreover, berberine-loaded disulfide-bridged mesoporous organosilica nanoparticles (ss-MONs) could enhance its accumulation in liver cancer tissue, avoid rapid blood clearance, and surmount several other obstacles from using berberine alone, such as poor gastrointestinal absorption and poor targeting abilities [131]. In addition, PEG-PE/TPGS-mixed micelles, which was made of 1,2-distearoyl-sn-glycero-3-phospho-ethanolamine-N-(methoxy(polyethyleneglycol)2000) (PEG-PE) mixed with d-a-tocopheryl polyethylene glycol 1000 succinate (TPGS) in a 3:1 M ratio, could increase berberine solubilization by 300% and enhance the cytotoxic effectiveness of berberine against prostate cancer cells [132]. Furthermore, berberine-loaded selenium nanoparticles (SeNPs) were more easily taken up by cells because of the nano-size of the SeNPs [133].

## 5. Safety of Berberine

Several studies showed that berberine had low toxic effects on human beings. For example, a phase I clinical trial showed that berberine was safe at excessive doses [134]. Another study found that berberine showed low toxicity towards healthy cells [135]. Although berberine could contribute to some adverse events, such as constipation and nausea, these adverse effects are not serious. The most common event constipation disappeared once berberine was discontinued [136,137].

## 6. Conclusions and Perspectives

Cancer is a severe public health problem in the world. Berberine showed anticancer activities in various cancers, such as breast, lung, gastric, liver, colorectal, prostate, and ovarian cancers. The potential mechanisms include inhibiting cell proliferation, suppressing metastasis, inducing cell apoptosis, activating autophagy, regulating gut microbiota, and enhancing the effects of other antitumor drugs. Moreover, different nanomaterials have been developed, such as AgNPs, ss-MONs, and SeNPs, in order to improve berberine bioavailability and enhance its anticancer activities. In addition, berberine is safe and tolerable for human beings, although some side effects were observed, such as constipation and nausea. In the future, effects of berberine against more cancers should be evaluated, and the relative mechanisms should be studied. Furthermore, the effects and mechanisms of berberine inhibiting migration of cancers should be further explored from the perspective of intratumoral microbes inhibition or gut microbiota regulation. Additionally, more methods or techniques should be developed to improve the bioavailability and anticancer activity of berberine. More clinical trials should be carried out to confirm effects of berberine on human beings, and adverse effects should also be observed.

## Figures and Tables

**Table 1:** Effects and mechanisms of berberine on several cancers.

Study Types	Models	Dosages	Effects and Mechanisms	Ref.
**Breast cancer**				
In vitro	MCF-7 and MDA-MB-231 cells	0, 1, 10, 50, 100, and 200 μM	Inhibit proliferative ability of breast cancer cells by reducing metadherin	[25]
In vitro	MCF7 and MCF12A cells	1, 10, and 100 μM	Induce the nucleolar stress response Upregulate the p53	[36]
In vitro	MCF-7 and MDA-MB-231 cells	25 and 50 µM	Upregulate miR-214-3p Reduce mRNA expression and protein levels of SCT	[32]
In vitro	MCF-7 and MDA-MB-231 cells	20, 40 and 80 µM	Upregulate p21/cip1 and p27/kip1 Increase nuclear localization and post-translational protein stability	[37]
In vitro	MDA-MB-468, MDA-MB-231, HCC70, HCC38, HCC1937, HCC1143, BT-20, and BT-549 cells	0.5 and 1 µM	Induce cell cycle arrest Induce cell apoptosis	[29]
In vitro	MDA-MB-231 cells	2.5, 5, 10, 20, 40, 60, 80, 100 μg/mL	Reduce cell viability Increase LDH release Reduce the secretion of TNF-α, IL-1α, IL-1β, and IL-6	[26]
In vitro	MDA-MB-231, MDA-MB-468, MDA-MB-453, and BT-549 cells	MDA-MB-231: 0, 6.25, 12.5, and 25 µM; MDA-MB-468: 0, 3, 6, and 12 µM; MDA-MB-453: 0, 2.5, 5, and 10 µM; BT-549 cells: 0, 5, 10, and 20 µM	Inhibit cell proliferation Induce cell cycle arrest Reduce the expression of cyclin A and CDK1 Reduce cyclin D and CDK4 expression	[30]
In vitro	MDA-MB-453, BT20, BT549, MDA-MB-231, Hs578T, and MDA-MB-157 cells	50 µM	Decrease fibronectin expression through inhibition of AP-1 activity	[33]
In vitro	Canine mammary gland carcinoma cell line	10, 25, 50, 100 and 200 µM	Inhibit cell proliferation Decrease cell viability	[28]
In vitro	MCF-7/MDR cells	5, 10, 20 μmol/L	Induce cell apoptosis via AMPK-p53 signaling pathway	[35]
In vitro	MCF-7 cells	10, 20, 40 and 80 μg/mL	Decrease cell migration through downregulation of several chemokine receptors	[31]
In vitro	MCF-7, T47D, MDA-MB-468, and MDA-MB-231 cells	Berberine: 0–40 µM; emodin: 0–40 µM	Inhibit cell growth via inhibiting SIK3 activity Induce G_0_/G_1_ phase cell cycle arrest and apoptosis	[39]
**Colorectal cancer**				
In vitro	HCT116 cells	1, 10 and 100 µM	Regulate the three-gene network miR21-ITGβ4-PDCD4	[90]
In vitro	CACO2 and LOVO CRC cell lines	0, 10, 20, 40, 60, 80 µM	Inhibit mitochondrial protein synthesis, TCA, and respiratory electron transportation	[125]
In vitro	SW480 and HT-29 cells	0, 20, 50, 100, 200, and 300 µM	Inhibit GRP78 expression Upregulate cytokeratin expression.	[89]
In vitro	SW480 and HT-29 cells	0, 25, 50, 100, 200, 400 and 800 µM	Inhibit cell proliferation Induce cell cycle arrest at G0/G1 phase	[87]
In vitro	HT29 and HCT116 cells	0, 10, 20, 40, 60, 80, 100 µM	Induce cell apoptosis via modulation of lncRNA CASC2/AUF1/Bcl-2 axis	[91]
In vitro	HCT 116 cells	10.54 µg/mL	Decrease the activity and the level of telomerase Inhibit cell proliferation	[85]
In vitro	DLD-1 and Caco-2 cells	6.25, 12.5, 25, 50 µM	Induce cell cycle arrest at G0/G1 phase Modulate lipogenesis through targeting the SCAP/SREBP-1 pathway	[86]
In vitro	KM12C cell	6.25, 12.5, 25, 50 µM	Suppress β-catenin function through binding RXRα Inhibit cell proliferation	[83]
In vivo	BALB/c nude mice	10 mg/kg	Inhibit the xenograft tumor growth	[83]
In vitro	Colorectal cancer tissues	4, 8, 16 µM	Downregulate miR-429 expression	[126]
In vitro	HCT116 and HT29 cells	10, 20, and 40 μM	Induce cancer cell apoptosis Decrease m^6^A methylation via decreasing β-catenin and increasing FTO Inhibit cell proliferation via decreasing cyclin D1, increasing p27 and p21 Induce cell cycle arrest at G_1_/G_0_ phase	[81]
In vivo	BALB/c nude mice	5, 10, or 20 mg/kg	Inhibit the tumor growth	[81]
In vitro	HT-29, HCT116, and SW620 cell lines	2, 10 and 50 μM	Inhibit cell proliferation via regulating β-catenin	[82]
In vivo	C57BL/6 nude mice	5 and 50 mg/kg	Increase the survival rates Decrease β-catenin expression Inhibit the tumor growth of tumor-bearing mice	[82]
In vivo	C57BL/6 male mice	7.5 and 15 mg/kg	Inhibit the development of precancerous lesions Improve intestinal barrier function Increase the ratio of *Firmicutes/Bacteroidetes* Decrease the abundance of cancer-related bacteria	[94]
In vitro	HCT-8, HCT-116, and HT-29 cells	6.25, 12.5, 25, 50 μM	Suppress lipogenesis via promotion of PLZF-mediated SCAP ubiquitination Inhibit colon cancer cell metastasis	[88]
**Gastric cancer**				
In vitro	AGS and HGC27 GC cells	0, 20, 50, 80 μM	Decrease cell viability Inhibit cell proliferation Induce cell apoptosis Modulate circRNA expression and their corresponding target genes	[68]
In vitro	SGC-7901 cells	2.5, 5, 10, 20, and 30 µM	Inhibit cell proliferation; Induce cell cycle arrest at G1 phase; Induce apoptosis Induce autophagy	[64]
In vitro	BGC-823 cells	14, 21, 32, 48, 72, and 108 μM	Induce cytostatic autophagy via inhibition of mTOR, Akt, and MAPK (ERK, JNK, and p38) pathways	[67]
In vivo	BALB/c nude mice	5, 10, 20 mg/kg	Induce cytostatic autophagy via inhibition of mTOR, Akt, and MAPK pathways	[67]
In vitro	SGC-7901, BGC-823, SGC-7901/DDP, and BGC-823/DDP cells	Berberine:10 μM; cisplatin: 2, 4, 8, 16, 32, 64 μg/mL	Increase cisplatin sensitivity cancer cells by upregulating miR-203 expression	[70]
In vitro	MKN45, BGC823, and SGC7901 cells	Berberine: 15 to 90 μM; cetuximab: 0.03, 0.06, 0.13, 0.25, 0.50, 1.00, 2.00 mg/mL	Inhibit the activation of STAT3 via inhibiting the phosphorylation of EGFR Downregulate the expression of Bcl-xL and cyclin D1 Synergistic effect with erlotinib and cetuximab	[66]
In vivo	BALB/C-nu/nu nude mice	Berberine: 50 mg/kg; cetuximab: 0.8 mg/mouse/ day	Enhance the growth inhibitory activity of cetuximab Inhibit EGFR signaling	[66]
In vitro	MGC 803 cells	0, 7.5, 15, 30 and 60 µM	Inhibit cell proliferation Reduce IL-8 secretion Deactivate MAPK signaling pathway	[65]
In vivo	BALB/C nude mice	15 mg/kg	Reduce tumor weight and volume Reduce IL-8 secretion	[65]
**Hepatic cancer**				
In vitro	Hep3B and BEL-7404 cells	12.5, 25, 50, 75, 100 and 125 μM	Suppress cell proliferation by inhibiting glutamine uptake via suppressing SLC1A5	[73]
In vivo	BALB/C nude mice	20 mg/kg	Suppress xenografts tumor growth Reduce SLC1A5 expression	[73]
In vitro	HepG2 and HUVEC cells	0.0625 to 8 mg/mL	Inhibit cell proliferation Induce cell cycle arrest at G_1_ and S phase Upregulate the intracellular ROS level Downregulate mitochondrial membrane potential	[74]
**Lung cancer**				
In vitro	A549, PC9, H460, H1299, Beas-2b, and 293T cells	0, 20, 40, 80, 120, and 160 μM	Promote cell apoptosis through miR19a/TF/MAPK signaling pathway	[58]
In vitro	A549, H1299, and H1975 cells	0, 60, 120 μmol/L	Inhibit cancer cell growth via suppressing DNA repair and replication	[57]
In vivo	C57BL/6 mice	200 mg/kg	Enlarge tumor necrosis area	[57]
In vitro	A549 cells	0, 30, 60, 90, 150 and 200 µM	Inhibit cell proliferation through MMP-2, Bcl-2/Bax and Jak2/VEGF/NF-κB/AP-1 signaling pathways	[55]
In vitro	NCI-H460, A549 and NCI-H1299 cells	10, 20, 40 and 80 µM	Suppress the proliferation and colony formation of cancer cells; Induce cell apoptosis by inducing DNA damage via downregulating the level of TOP2β	[54]
In vivo	BALB/c nude mice	25 mg/kg	Suppress tumor growth by deregulating Sin3A/TOP2β pathway	[54]
In vitro	EGFRm NSCLC cell lines and their derived resistant cell lines	Berberine: 12.5, 25, 50, 100, 200 μM; Osimertinib: 31.25, 62.5, 125, 250, 500 nM	Help osimertinib overcoming the acquired resistance caused by MET gene amplification	[15]
In vivo	nu/nu nude mice	Berberine: 25 mg/kg; osimertinib: 5 mg/kg	Enhance inhibitory activity against the growth of MET-amplified osimertinib-resistant tumors	[15]
In vitro	H1299 and A549 cells	Berberine: 25 and 50 μM; *P. amurense* extract: 2.5 and 5 μg/mL	Arrest cell cycle at G_1_ phase via Akt/CREB signaling axis Suppress cell proliferation via inhibiting proliferative kinase signaling	[56]
In vivo	Athymic nude mice	Berberine: 1000 or 1800 ppm; *P. amurense* extract: 3000 or 5400 ppm	Inhibit tumor growth	[56]
In vitro	A549 and PC9 cells	0, 40, and 80 µM	Induce cell apoptosis via activation of the ROS/ASK1/JNK pathway	[59]
**Ovarian cancer**				
In vitro	MDAH-2774 and SKOV-3 cells	0, 25, 50, 75 μM	Consume EGFR and ERBB2 in ovarian cancer cells; Inhibit the activation of EGFR and ERBB2 downstream targets cyclin D1, MMPs, and VEGF via EGFR-ERBB2/PI3K/Akt signaling pathway	[100]
In vitro	SKOV3 and 3AO cells	SKOV3: 40 μM; 3AO: 80 μM	Suppress Warburg effect by increasing TET3-related demethylation and upregulating miR-145	[97]
In vitro	SKOV3 cells	Bebrerine: 5 μmol/L; VP16: 5 μmol/L	Reverse chemotherapy drug VP16 induced repopulation of ovarian cancer cells by blocking the iPLA2-AA-COX-2-PGE_2_ pathway Reverse the increased phosphorylation of FAK	[102]
In vitro	A2780, HEY, SKOV3, FTE-187, HO8910, and OVCAR3 cells	5, 10, 20 μM	Sensitize cancer cells to PARP inhibitors Induce oxidative DNA damage Downregulate HRR	[103]
**Cervical cancer**				
In vitro	Ca Ski cells	0, 50, 100, 150 µM	Increase GADD153 expression by inducing ROS production; Induce mitochondria dysfunction; Activate caspase-3 and cytochrome C release	[127]
In vitro	SiHa, HeLa, and CaSki cells	5, 10, 15, 20 µM	Reduce cell invasion and migration; Reduce transcriptional activities of MMP-2 and u-PA; Reverse EMT via upregulating E-cadherin and inhibiting N-cadherin and snail-1 Reduce angiogenesis by downregulating VEGF	[106]
In vivo	BALB/c nude mice	20 mg/kg	Inhibit tumor growth Reduce tumor-induced angiogenesis	[106]
In vitro	HeLa and SiHa cells	3, 10, 30, 100, and 300 µmol/L	Inhibit cell proliferation Trigger cell apoptosis Induce cell cycle arrest at G1 phase	[107]
In vitro	Hela cells	0.098, 0.195, 0.391, 0.781, 1.563, 3.125, 6.25, 12.5, 25, and 50 µM	Overcome the radio-resistance Regulate glucose metabolism via PI3K/HIF-1 pathway	[108]
**Prostate cancer**				
In vitro	AIZ-AR cells	0.01–50 µM; 0.001–1000 nM	Induce cell apoptosis Inhibit cell proliferation	[111]
In vitro	22RV1 cell	1, 2.5, 5, 10, 20, 50 μM	Inhibit cell proliferation Induce cell apoptosis Downregulate the expression of AR, PSA, COX-2, and Bcl-2	[110]
In vivo	BALB/c nude mice	0.01136g/kg	Inhibit xenograft tumor growth	[110]
In vitro	LNCaP, PC3, PC3M, and 22RV1 cells	12.5, 25, 50 μmol/L	Suppress the intracellular androgen synthesis via inhibiting the AKR1C3 enzyme activity	[112]
In vitro	LNCaP and PC-3 cells	20, 100 and 200 μM	Arrest cell cycle at G1 phase Inhibit cell growth via inhibiting the activation of EGFR Induce cell apoptosis	[113]
In vitro	PC-3 and LNCaP cells	10, 25, 50, 75 μM	Inhibit cell invasion and migration by downregulating EMT-related genes	[114]
In vitro	LNCaP and DU-145 cells	20, 50, 100, 150, 200, 250, 300, 400 μM	Increase radiosensitivity cancer cells through inhibiting the expression of HIF-1α and VEGF	[115]
**Bladder cancer**				
In vitro	T24, 5637, SV-HUC-1 cells	20, 40, 60 µM	Disturb AK2-STAT3 signaling pathway via up-regulating miR-17-5p	[19]
In vivo	BALB/c nude mice	200 mg/kg	Promote miR-17-5p expression Suppress JAK1 and STAT3	[19]
In vitro	T24 and 5637 cells	1, 5, 10, 20, 40, 80, 160 µM	Enhance gemcitabine-induced cytotoxicity	[116]
**Endometrial cancer**				
In vitro	AN3 CA and HEC-1-A cells	0, 10, 20, 40, 80, 160 µM	Inhibit cell progression and migration via miR-101/COX-2/PGE2 signaling pathway	[118]
In vivo	nude mice	50 mg/kg or 100 mg/kg	Inhibit cell invasion and migration	[118]
**Pancreatic cancer**				
In vitro	Panc-1 and hTERT-HPNE cells	2.5, 3.75, 5, 10 μM	Inhibit cell viability and migration by regulating citrate metabolism and transportation	[119]
**Hematopoietic Cancer**				
In vitro	HL-60, HL-60/MX1, HL-60/MX2, CCRF/CEM, CEM/C1, J45.01, and U266B1 cells	40–160 μM	Stimulate cell apoptosis	[120]
**Esophageal cancer**				
In vitro	KYSE-70 and SKGT4 cells	20, 40, 60 and 80 μmol/L	Arrest cell cycle at G_2_ phase Induce cell apoptosis,	[121]
In vitro	Eca9706, TE-1, and EC109 cells	Berberine: 90 μM; galangin: 30 μM	Synergistically inhibit cell growth Synergistically arrest cell cycle at G_2_/M phase Synergistically induce cell apoptosis	[122]
**Neuroblastoma**				
In vitro	N2a cells	0–20 µg/mL	Inhibit cancer stemness; Reverse the EMT	[123]
**Osteosarcoma**				
In vitro	MG-63 and HBMSC cells	Berberine: 2.5, 5, or 10 μM; cisplatin: 0, 1.25, 2.5, 5, or 10 μM	Induce apoptosis and cell cycle arrest at G_0_/G_1_ phase Inhibit MMP-2/9, Bcl-2, cyclin D1, and CDK4 expression Enhance Bax expression Regulate MAPK pathway	[124]

Abbreviation: AA, arachidonic acid; AKR1C3, aldo-keto reductase family 1 member C3; Akt, protein kinase B; AP-1, activator protein 1; AR, androgen receptor; AUF1, AU-biding factor 1; ASK1, apoptosis signal-regulating kinase 1; Bax, Bcl-2-associated X protein; Bcl-2, B-cell lymphoma 2; BLE: *Berberis lyceum* extracts; CASC2, cancer susceptibility candidate 2; CDK, cyclin-dependent kinase; circRNA, circular RNA; COX-2, cyclooxygenase-2; CRC, colorectal cancer; EGFR, also known as ERBB, epidermal growth factor receptor; EMT, epithelial-mesenchymal transition; FAK, Focal adhesion kinase; GC, gastric cancer; GRP78, glucose-regulated protein 78; HIF-1α, hypoxia inducible factor-1α; HRR, homologous recombination repair; IL-1α, interleukin-1α; IL-1β, interleukin-1β; IL-6, interleukin-6; IL-8, interleukin-8; iPLA_2_, independent phospholipase A_2_: ITGβ4, integrinβ4; Jak, Janus kinase; KRT, keratin; JNK, c-Jun N-terminal kinase; LC3, microtubule-associated protein light chain 3; LDH, Lactate dehydrogenase; lncRNA, long non-coding RNA; miR, microRNA; mTOR, mammalian target of rapamycin; MMP-2, matrix metalloproteinase 2; NF-κB, nuclear factor κB; NSCLC, non-small cell lung cancer; N2a, neuro2a; *P. amurense*, *Phellodendron amurense*; PDCD4, programmed cell death 4; PGE_2_, prostaglandin E_2_; ppm, parts per million; PLZF, promyelocytic leukaemia zinc finger; PI3K, phosphatidylinositol-3-kinase; PSA, serum prostate-specific antigen; RXRα, retinoid X receptor α; SIK3, salt-inducible kinases 3; SCT, secretin; SCAP, sterol-regulatory element-binding proteins cleavage-activating protein; Sin3A, SWI-independent-3 transcription regulator family member A; SREBP-1, sterol regulatory element-binding protein-1; STAT3, transcription 3; TCA, the citric acid; TF, tissue factor; TNBC, triple-negative breast cancer; TNF-α, tumor necrosis factor-α; TOP2β, topoisomerase II β; u-PA, urokinase-type plasminogen activator; VEGF, vascular endothelial growth factor; XRCC1, X-ray cross complementing group 1 protein.
